# Plasma Free Amino Acid Responses to Whey Protein and Their Relationships with Gastric Emptying, Blood Glucose- and Appetite-Regulatory Hormones and Energy Intake in Lean Healthy Men

**DOI:** 10.3390/nu11102465

**Published:** 2019-10-15

**Authors:** Rachel A. Elovaris, Amy T. Hutchison, Kylie Lange, Michael Horowitz, Christine Feinle-Bisset, Natalie D. Luscombe-Marsh

**Affiliations:** 1 Adelaide Medical School and National Health and Medical Research Council of Australia Centre of Research Excellence in Translating Nutritional Science to Good Health, Adelaide Health and Medical Sciences Building, Corner North Terrace and George Street, Adelaide 5005, Australia; rachel.elovaris@adelaide.edu.au (R.A.E.); amy.hutchison@adelaide.edu.au (A.T.H.); kylie.lange@adelaide.edu.au (K.L.); michael.horowitz@adelaide.edu.au (M.H.); christine.feinle@adelaide.edu.au (C.F.-B.); 2 Nutrition and Metabolism Theme, South Australian Health and Medical Research Institute, Adelaide 5000, Australia; 3 Commonwealth Scientific and Industrial Research Organisation (CSIRO), Nutrition and Health Program, P.O. Box 10097, Adelaide 5000, Australia

**Keywords:** branched-chain amino acids, dairy, appetite regulation, cholecystokinin, glucagon-like peptide-1, glucagon, human

## Abstract

This study determined the effects of increasing loads of whey protein on plasma amino acid (AA) concentrations, and their relationships with gastric emptying, blood glucose- and appetite-regulatory hormones, blood glucose and energy intake. Eighteen healthy lean men participated in a double-blinded study, in which they consumed, on 3 separate occasions, in randomised order, 450-mL drinks containing either 30 g (L) or 70 g (H) of pure whey protein isolate, or control with 0 g of protein (C). Gastric emptying, serum concentrations of AAs, ghrelin, cholecystokinin (CCK), glucagon-like-peptide 1 (GLP-1), insulin, glucagon and blood glucose were measured before and after the drinks over 180 min. Then energy intake was quantified. All AAs were increased, and 7/20 AAs were increased more by H than L. Incremental areas under the curve (iAUC_0–180 min_) for CCK, GLP-1, insulin and glucagon were correlated positively with iAUCs of 19/20 AAs (*p* < 0.05). The strongest correlations were with the branched-chain AAs as well as lysine, tyrosine, methionine, tryptophan, and aspartic acid (all R^2^ > 0.52, *p* < 0.05). Blood glucose did not correlate with any AA (all *p* > 0.05). Ghrelin and energy intake correlated inversely, but only weakly, with 15/20 AAs (all R^2^ < 0.34, *p* < 0.05). There is a strong relationship between gluco-regulatory hormones with a number of (predominantly essential) AAs. However, the factors mediating the effects of protein on blood glucose and energy intake are likely to be multifactorial.

## 1. Introduction

High-protein diets, including those that incorporate about 2–3 serves of dairy protein, are effective in the management of obesity and associated cardio-metabolic conditions [1,2,3,4,5]. As one of the main components of dairy, whey is common in the diet and, when compared with other sources of protein, has been shown to be more satiating and more effective in facilitating weight loss [4,6,7].

The importance of gastrointestinal (GI) mechanisms to the beneficial effects of foods and beverages rich in dietary protein, particularly whey, on weight loss and cardio-metabolic functions has been well established over the last decade [4,5,8,9]. Several studies have reported dose-dependent effects of whey protein on concentrations of blood glucose- and appetite-regulatory hormones, including cholecystokinin (CCK), glucagon-like peptide-1 (GLP-1), insulin and glucagon [10,11,12,13,14,15]. In healthy individuals, whey protein in loads of 4.5–18 g (in a drink containing 25 g glucose) dose-dependently lowered postprandial glycaemia and increased insulin, with a minimum of 9 g required for a significant effect [8]. Furthermore, in healthy young adults drinks containing whey protein isolate loads of 20 g, 30 g or 40 g (consumed 30 min before a standardised carbohydrate-rich pizza meal) suppressed energy intake and reduced post-meal blood glucose more than 0 or 10 g loads [9].

Specific amino acids (AAs) that reach the peripheral circulation are purported to be integral to the modulation of blood glucose- and appetite-regulatory hormones by protein-rich foods/beverages [13,14]. In healthy individuals, intraduodenal (ID) administration of whey protein in loads of 2.1, 6.3, and 12.5 kcal/min (to mimic the range of normal gastric emptying rates of protein of 1–4 kcal/min) over 60 min increased plasma concentrations of 19 out of 20 AAs (the exception being cysteine) in a protein load-dependent manner [16]. Moreover, the increases in the branched-chain and essential (methionine, lysine and tyrosine) AAs were strongly, and positively, related with GLP-1 and insulin, and moderately, and inversely, with energy intake [16]. In another study, in which healthy men consumed drinks with 30 or 70 g of whey protein, the amount of calories emptied from the stomach by 60 min was associated significantly, albeit modestly, with the magnitude of change over the first 60 min in ghrelin, CCK, GLP-1 and glucagon concentrations (all r values > 0.5, *p* < 0.05), and these gut hormones were each modestly associated (inversely in the case of ghrelin) with the concordant suppression of energy intake (~11% suppression) by both protein drinks [17]. There is no information about the relationships between the release of specific AAs with glucose- and appetite-regulatory hormones, gastric emptying and energy intake responses following the consumption of protein-enriched drinks.

We have now analysed remaining plasma samples from our previous study [17] to evaluate the hypothesis that oral whey protein loads of 30 and 70 g would lead to load-dependent rises in specific AAs, and that the effects of protein on gastric emptying, blood glucose- and appetite-regulatory hormones and energy intake would be related to circulating concentrations of specific branched-chain and other essential AAs.

## 2. Materials and Methods

### 2.1. Participants

Twenty lean, healthy men (mean age 24.7 ± 1.2 years [range 18–37 years]; mean BMI 22.0 ± 0.5 kg/m^2^ [range 18.6–25.0 kg/m^2^]) were recruited into the study as described [17]. All participants provided written, informed consent to participate in the study, which was approved by the Research Ethics Committee of the Central Adelaide Local Health Network. The number of participants was determined from power calculations on the basis of our previous work, indicating that *n* = 16 participants would allow detection of a mean difference of 20.5 min in gastric 50% emptying time (T_50_), while *n* = 20 participants would allow detection of a mean difference in energy intake between treatments of 215 kcal, with β = 0.8 and α = 0.05 [18,19]. Only males were studied, due to known variations in energy intake across the menstrual cycle in females [20]. Participants who were identified as restrained eaters (score ≥ 12 on the eating restraint component of the Three-Factor Eating Questionnaire) [21], had low ferritin (<30 ug/L) or iron (<8 umol/L) concentrations, were lactose-intolerant, vegetarians, or were high-performance athletes, were excluded from participating. The Royal Adelaide Hospital Research Ethics Committee approved the study protocol, and the study was registered as a clinical trial with the Australia and New Zealand Clinical Trial Registry (www.anzctr.org.au, registration number 12611000706976).

### 2.2. Study Outline

The aims of the original study were to evaluate the effects of 450-mL drinks containing 30 g pure whey protein isolate (L), 70 g pure whey protein isolate (H), or 0 g (control) on gastric emptying, GI hormone release, plasma insulin, glucagon, total AAs, blood glucose, appetite and energy intake [17]. Accordingly, the evaluation of the effects of the drinks on the temporal release of all 20 AAs, and relationships with the previously reported outcomes, represents an exploratory secondary analysis.

### 2.3. Protein Drinks

As described [17], the pure protein drinks (i.e., they did not contain any other macronutrients) were prepared in the morning of each study visit by a member of the research staff, who had no involvement in either the analysis or interpretation of the data. Whey protein isolate powder (8855 ClearPro, Fonterra Co-Operative Group Ltd., Auckland, New Zealand) was dissolved in distilled water and diet cordial (Bickford’s Diet Lime Cordial, Bickford’s Australia) to achieve the desired loads (i.e., L-30 g whey protein (total energy content = 126 kcal) or H-70 g whey protein (total energy content = 283 kcal). The control drink consisted of 90 mL cordial and 359 mL distilled water (total energy content = 11.5 kcal). Sodium chloride was added to the L and C drinks in amounts of 0.3 g and 1.2 g, respectively, to match the osmolarity with H (i.e., 88 mOsmol/L) because it is well-established that osmolarity of liquid solutions affects gastric emptying [22,23]. The drink was provided to each participant in an opaque cup, covered at all times, so that both the primary investigator and the participant were blinded to the treatment, and consumed within 2 min. Table A1 outlines the AA composition of the whey protein isolate, and the amounts present in each drink.

### 2.4. Protocol

Each participant was studied on three occasions, separated by 7–11 days, in a randomised, double-blind, cross-over design [24]. Randomisation and preparation of the solutions were performed by an investigator who had no involvement in the studies or data analysis. Participants were provided with a standardised meal, consumed on the evening before each study day, and instructed to abstain from all food, drinks and vigorous exercise until attending the laboratory at the Discipline of Medicine at 0830 h. At t = −10 min, a 14-mL blood sample was collected, a visual analogue scale questionnaire (VAS) administered and a 3-dimensional (3D) image of the stomach, obtained using 3D ultrasound, was recorded. At t = −2 min, participants then ingested one of the test drinks. Immediately afterwards, at t = 0 min, and subsequently, at 15-min intervals, until t = 180 min, further 3D ultrasound images, blood samples and VASs were obtained. At t = 180 min, each participant was presented with a standardised, cold, buffet-style test meal, as described [18], and instructed to consume as much food until they felt comfortably full, for up to 30 min (t = 180–210 min).

### 2.5. Measurements

#### 2.5.1. Gastric Emptying

Gastric emptying was measured by 3D ultrasonography with the use of a Logiq 9 ultrasound system (GE Health Care Technologies, Milwaukee, WI, USA) with TruScan Architecture (a built-in magnetic sensor for 3D image acquisitions), as described [17].

#### 2.5.2. Plasma Ghrelin, CCK, GLP-1, Insulin, Glucagon, Free AA and Blood Glucose Concentrations

10-mL blood samples were collected into ice-chilled ethylenediaminetetraacetic acid-coated tubes. Blood samples were centrifuged immediately (3200 rpm for 15 min at 4 °C) to obtain plasma. Plasma samples were stored at −70 °C for subsequent analysis.

Plasma total ghrelin concentrations (pmol/L) were analysed by radioimmunoassay, as described [25], without peptide extraction (Phoenix Pharmaceuticals, Burlingame, CA, USA). No cross-reactions with relevant molecules have been evident. Detection limit was 1.0 mU/L, intra-assay and inter-assay coefficients of variation (CVs) were 7.0% and 13.4%, respectively.

Plasma CCK-8 concentrations (pmol/L) were analysed by radioimmunoassay after ethanol extraction, using an adaption of a previous method, as described [26]. Standards were prepared with the use of a synthetic sulfated CCK-8 antibody (Sigma Chemical, St. Louis, MO, USA) which binds all CCK peptides containing a sulfated tyrosine residue in position 7, shows a 26% cross-reactivity with unsulfated CCK-8, <2% cross-reactivity with human gastrin I, and does not bind to structurally unrelated peptides. Sulfated CCK-8 ^125^I-labeled with Bolton and Hunter reagent (Perkin Elmer) was used as a tracer, and samples were incubated for 7 days at 4 °C. The antibody-bound fraction was separated by the addition of dextran-coated charcoal containing gelatin and the radioactivity determined in the supernatants after centrifugation. The detection limit was 1 pmol/L, and intra-assay and inter-assay CVs were 8.4% and 16.5%, respectively.

Plasma GLP-1 concentrations (pmol/L) were analysed by radioimmunoassay (GLPIT-36HK; Millipore, Billerica, MA, USA) [27]. There are no cross-reactions with glucagon, gastric inhibitory polypeptide or other gut or pancreatic peptides, and it measures both GLP-1_(7–36)_ and GLP-1_(9–36)_ amide. The detection limit was 3 pmol/L, and intra- and inter-assay CVs were 7.1% and 7.8%, respectively.

Plasma insulin concentrations (mU/L) were analysed by ELISA (10-1113; Mercodia, Uppsala, Sweden) [27]. The detection limit was 1.0 mU/L, and intra- and inter-assay CVs were 2.8% and 8.2%, respectively.

Plasma glucagon concentrations (pmol/L) were analysed by radioimmunoassay (GL-32K; Millipore, Burlington, MA, USA). The antibody used does not cross-react with insulin, proinsulin, C-peptide, somatostatin, or pancreatic polypeptide, and has <0.1% cross-reactivity with oxyntomodulin. The detection limit was 6 pmol/L, and intra-assay and inter-assay CVs were 4.2% and 9.3%, respectively.

Plasma free AA concentrations (mmol/L) for aspartic acid, alanine, arginine, asparagine, cysteine, glutamic acid, glutamine, glycine, histidine, isoleucine, leucine, lysine, methionine, phenylalanine, proline, serine, threonine, tryptophan, tyrosine and valine were measured, as described [16]. The analysis was performed at the Australian Proteome Analysis Facility established under the Australian Government’s National Collaborative Research Infrastructure Strategy.

Blood glucose concentrations (mmol/L) were measured immediately after collection, by the glucose oxidase method using a portable glucometer (FreeStyle Optimum H; Abbott Laboratories, Chicago, IL, USA).

#### 2.5.3. Energy Intake

Each food item in the buffet meal [19] was weighed before and after consumption to quantify the amounts of food and beverages consumed (g). Energy intake (kcal) was then calculated using commercially available software (Foodworks 3.01, Xyris Software, Highgate Hill, QLD, Australia) [19].

### 2.6. Data and Statistical Analysis

Statistical analysis was performed using SPSS software (version 24; IBM, Armonk, NY, USA), in consultation with a biostatistician. Baseline plasma AA concentrations (i.e., t = −2 min) between study days were analysed using one-way repeated measures ANOVA with protein load as the factor. The effects of protein load on the net incremental area under the curve (iAUC_0–180 min_) for each AA were analysed by general linear model mixed model ANOVA. Two-way repeated measures ANOVA (treatment-by-time model) was conducted, with post-hoc pairwise comparisons at individual time points. Post-hoc comparisons, adjusted for multiple comparisons by Bonferroni correction, were performed when significant treatment, or treatment-by-time, effects, were found. To visually present the plasma AAs that had the “smallest” (bottom 5) and “greatest” (top 5) responses to the test drinks, we defined the response based on the magnitude of increase in each plasma AA concentration over the 180-min period following the L and H drink, respectively, and relative to C. The formula below was used to calculate the magnitude of increase in each plasma AA:

Magnitude increase (expressed as a %) = iAUC_0–180 min_ for each specific AA following H, divided by iAUC_0–180 min_ for each specific AA following C, multiplied by 100.

Relationships between the iAUC for each AA and protein load, total AA concentration within the protein drinks, gastric emptying (half-emptying time, T_50_) [17], energy intake and iAUCs for ghrelin, CCK, GLP-1, insulin, glucagon and blood glucose, respectively, were evaluated using linear within-subject correlations (*r*) with fixed slopes and subject-varying intercepts [28]. Relationships of each AA with gastric emptying, energy intake and iAUCs for ghrelin, CCK, GLP-1, insulin, glucagon and blood glucose, respectively, were ranked in order of strongest to weakest response.

Statistical significance was accepted at *p* < 0.05. All data are reported as means ± SEMs.

## 3. Results

Twenty participants were recruited for the study. Data from 16 participants who completed all study days, who tolerated the drink and for whom no data were missing, was analysed. Reasons for excluding the data from 4 participants were failure to fast overnight (*n* = 1), completion of only 2 of the 3 study days before withdrawing due to time constraints (*n* = 1), and exclusion of gastric emptying data due to suboptimal image quality (i.e., presence of air in the stomach) (*n* = 2). There were no differences in the characteristics of the 16 completers compared with the 4 excluded participants, and no exclusion was related to the protein drinks.

### 3.1. Plasma AA Concentrations in Response to Increasing Protein Loads

Baseline concentrations of individual and total AAs did not differ between test days with the exception of tryptophan (*p* < 0.05) (Table 1).

**Figure 1:**
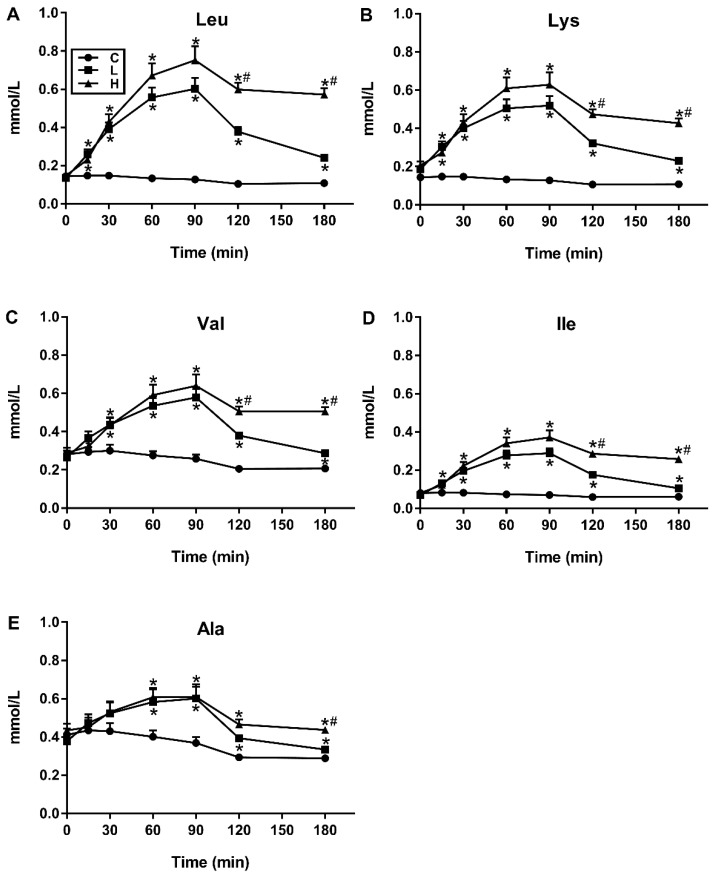
Temporal profiles of (**A**) Leucine (Leu), (**B**) Lysine (Lys), (**C**) Valine (Val), (**D**) Isoleucine (Ile) and (**E**) Alanine (Ala), the five amino acids whose plasma concentrations increased the most in response to test drinks containing either 0 g (C), 30 g (L) or 70 g (H) of pure whey protein dissolved in varying amounts of distilled water, diet cordial, and sodium chloride so they were matched for volume and osmolarity (all 450 mL and 88 mOsm/L). These responses were defined as being the strongest based on the “magnitude of increase” in each plasma concentration over the 180-min period following the L and H drink, respectively, and relative to C. Data are means ± SEMs, *n* = 16. Effects of protein load and time on individual AAs were determined by a two-way ANOVA, and post-hoc comparisons between two loads were determined using Bonferroni’s correction; statistical significance was accepted at *p* < 0.05. * Significantly different from C; # Significantly different from L (*p* < 0.05).

**Figure 2:**
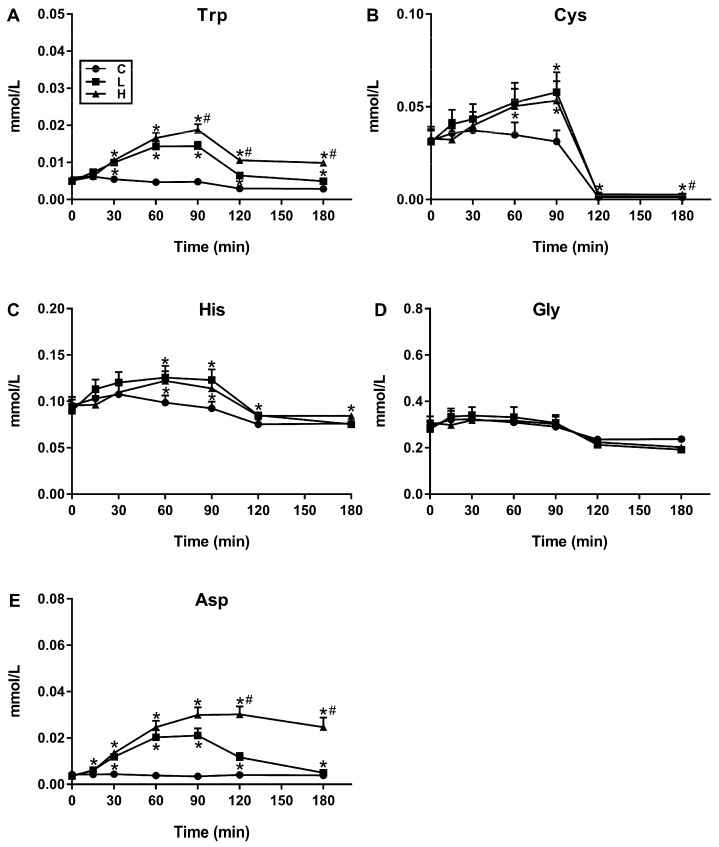
Temporal profiles of (**A**) Tryptophan (Trp), (**B**) Cysteine (Cys), (**C**) Histidine (His), (**D**) Glycine (Gly) and (**E**) Aspartic Acid (Asp), the five amino acids with the weakest response to test drinks containing either 0 g (C), 30 g of whey protein (L) or 70 g of pure whey protein (H) dissolved in varying amounts of distilled water, diet cordial and sodium chloride so they were matched for volume and osmolarity (all 450 mL and 88 mOsm/L). These responses were defined as being the weakest based on the “magnitude of increase” in each plasma concentration over the 180-min period following the L and H drink, respectively, and relative to C. Data are means ± SEMs, *n* = 16. Effects of protein load and time on individual AAs were determined by a two-way ANOVA, and post-hoc comparisons between two loads were determined using Bonferroni’s correction; statistical significance was accepted at *p* < 0.05. * Significantly different from C; # Significantly different from L (*p* < 0.05).

There was a significant treatment-by-time interaction for plasma concentrations of 20/20 AAs (*p* < 0.05 for all). The greatest increases in plasma concentrations over the 180-min period following L and H, relative to C, were for leucine, lysine, valine, isoleucine and alanine (Figure 1), all of which diminished after 90 min, although more slowly after H than L. Conversely, smallest increases were evident for tryptophan, cysteine, histidine, glycine and aspartic acid (Figure 2).

The iAUC_0–180 min_ of each of the 20 AA profiles are depicted in Table 2. There was a significant main effect of protein load on the iAUC_0–180 min_ of plasma concentrations of 19 out of 20 AAs (exception: glycine) (*p* < 0.05 for all). Post-hoc analyses revealed that the iAUC_0–180 min_ of plasma concentrations of 19 out of 20 AAs (*p* < 0.05 for all) (exception: glycine) were greater in response to L compared with C. Furthermore, for 18 out of 20 AAs (*p* < 0.05 for all) (exceptions: glycine and glutamine) the responses to H were greater than to C. While plasma concentrations of 13 out of 20 AAs did not differ between L and H protein drinks, the iAUC_0–180 min_ of leucine, lysine, isoleucine, tyrosine, glutamic acid, methionine and aspartic acid were greater following H than L (*p* < 0.05 for all). Relationships between the iAUCs_0–180 min_ of each of the 20 AAs (displayed in order of abundance of the AAs within the whey protein drinks) with the load of protein are presented in Table A3. The magnitude of increase in the plasma concentrations of 8 out of 9 essential AAs (exception: histidine) was moderately to strongly associated with the concentration of the protein in the drinks (i.e., range of R^2^ values was 0.54–0.84, *p* < 0.05 for all). In contrast, the relationships for the non-essential AAs (i.e., range of R^2^ values was 0.26–0.83, *p* < 0.05 for all) and 5 out of 6 conditional AAs (exception: glycine for which there was no relationship) (i.e., range of R^2^ values was 0.14–0.72, *p* < 0.05 for all) were more variable.

### 3.2. Relationships between Gastric Emptying, Ghrelin, CCK, GLP-1, Insulin, Glucagon, Blood Glucose and Energy Intake with Plasma AA concentrations

For the purpose of the current exploratory relationships analysis, the temporal profiles of gastric emptying, and plasma ghrelin, CCK, GLP-1, insulin, glucagon, and blood glucose reported previously [17] have been expressed in Table A2.

**Figure 3:**
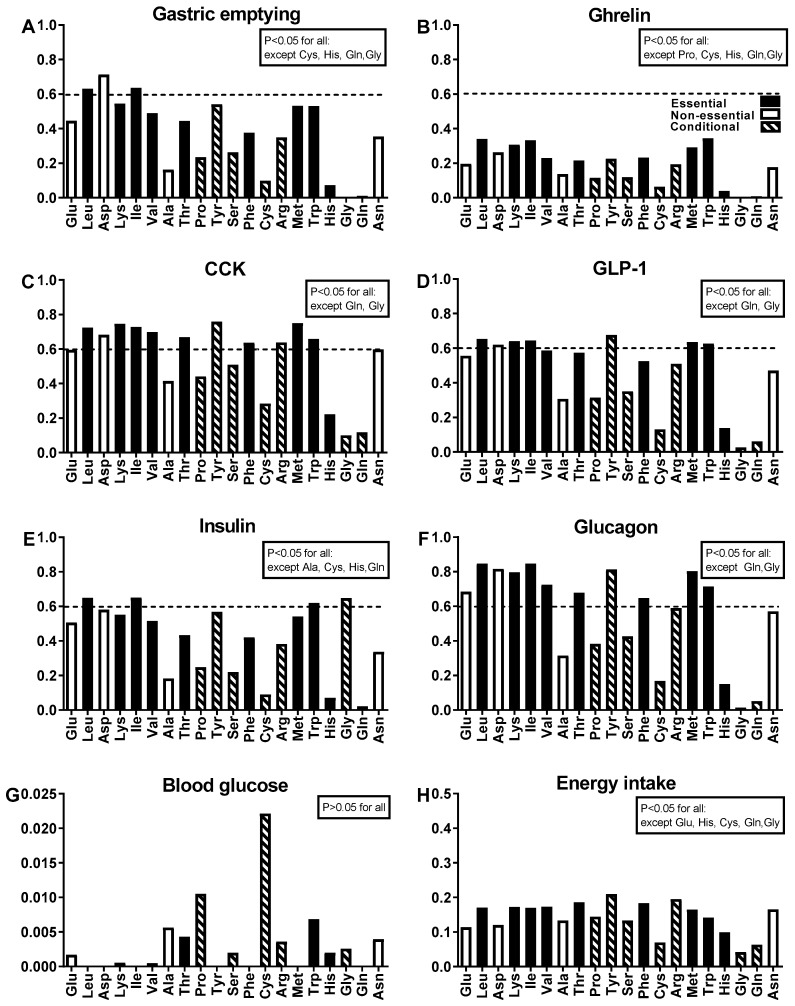
Within-subject relationships between the iAUCs_0–180 min_ of each of the 20 amino acids (displayed in order of abundance of the amino acids in the whey protein drinks) with (**A**) gastric emptying, as well as the iAUCs_0–180 min_ of (**B**) ghrelin, (**C**) CCK, (**D**) GLP-1, (**E**) insulin, (**F**) glucagon and (**G**) blood glucose and (**H**) energy intake, following consumption of the three test drinks which contained 0 g (C), 30 g (L), and 70 g (H) of whey protein. Data are within-subject R^2^ values for *n* = 16; Statistical significance was accepted at *p* < 0.05. The hatched horizontal line indicates the specific AAs which were consistently amongst the top ~10 amino acids strongly associated (defined as having an R^2^ of ≥0.6) with the majority of study outcomes presented in A to F except ghrelin. Abbreviations for AAs: Alanine: Ala; Arginine: Arg; Asparagine: Asn; Aspartic Acid: Asp; Cysteine: Cys; Glutamine: Gln; Glutamic acid: Glu; Glycine: Gly; Histidine: His; Isoleucine: Ile; Leucine: Leu; Lysine: Lys; Methionine: Met; Phenylalanine: Phe; Serine: Ser; Threonine: Thr; Tryptophan: Trp; Tyrosine: Tyr; Valine: Val.

The strength of the relationships between the iAUCs_0–180 min_ for each of the 20 amino acids with gastric emptying, ghrelin, CCK, GLP-1, insulin, glucagon, glucose and energy intake following consumption of the three test drinks is illustrated in Figure 3. There were positive correlations for gastric emptying with the iAUCs_0–180 min_ of 16 out of 20 AAs (R^2^ range 0.16–0.71, *p* < 0.05 for all). There were also positive correlations between: (1) the iAUC_0–180 min_ of ghrelin with the iAUCs_0–180 min_ of 15 out of 20 AAs (R^2^ range 0.12–0.34, *p* < 0.05 for all); (2) the iAUCs_0–180 min_ of both CCK and GLP-1 with the iAUCs_0–180 min_ of 18 out of 20 AAs (CCK: R^2^ range 0.22–0.76 and GLP-1: R^2^ range 0.13–0.68, *p* < 0.05 for all); (3) the iAUC_0–180 min_ of insulin with the iAUCs_0–180 min_ of 16 out of 20 AAs (R^2^ range 0.22–0.65, *p* < 0.05 for all); and, (4) the iAUC_0–180 min_ of glucagon with the iAUCs_0–180 min_ of 18 out of 20 AAs (R^2^ range 0.15–0.85, *p* < 0.05 for all). There was no significant relationship between any AA with blood glucose concentrations (all *p* > 0.05). Energy intake was correlated inversely with the iAUCs_0–180 min_ for 15 out of 20 AAs (R^2^ range 0.12–0.21, *p* < 0.05). The iAUCs_0–180 min_ of the branched chain AAs, the essential AAs (particularly lysine, methionine and tryptophan), and the non-essential or conditional AAs (particularly aspartic acid and tyrosine) were most strongly related with gastric emptying and all glucose- and appetite-regulatory hormones, and weakly correlated with energy intake.

## 4. Discussion

This study in healthy men expands existing knowledge relating to the interplay between specific AAs within foods containing an intact or hydrolysed protein and how they collectively influence the modulation of glucose- and appetite-regulatory hormones, as well as energy intake. We have established for the first time that 450 mL, isoosmolar test drinks containing either 30 or 70 g of pure protein (i.e., contained no other macronutrients) substantially increased the plasma AA responses (iAUC_0–180 min_) of only 7 (i.e., leucine, isoleucine, lysine, methionine, tyrosine, glutamic acid and aspartic acid) of the 20 AAs in a load-of-protein dependent manner. Confirming previous studies by others [29,30] and our group [16], we found that the magnitude of increase in postprandial plasma concentrations for most of the essential AAs, reflected their abundance within the protein drinks, whereas the relationships for the non-essential and conditionally AAs were more variable. We have also established that plasma CCK, GLP-1, insulin and glucagon responses to the protein drinks were all strongly and positively correlated with specific AAs, particularly the essential AAs, while ghrelin and energy intake were only weakly, and inversely, correlated with 16 and 15 out of 20 AAs, respectively. Conversely, the blood glucose response was not correlated with any AA.

Previously we have reported that the postprandial plasma concentrations of 19 out of 20 AAs were increased in a load-of-protein dependent manner when increasing loads of whey protein (i.e., 8, 24 and 48 g) were delivered into the duodenum at rates mimicking the normal range of gastric emptying (i.e., 1–4 kcal/min) [16]. In contrast, despite the protein loads within the current study being substantially greater (i.e., 30 and 70 g) than used in our previous ID study, we observed that only 4 of the 9 essential AAs (leucine, lysine, isoleucine, methionine), 1 of the 7 conditionally essential AAs (tyrosine), and 2 of the 4 non-essential AAs (glutamic and aspartic acids), were increased in a load-of-protein dependent manner. Furthermore, in the current study we observed positive correlations, albeit variable in strength, between the concentration of AAs in the plasma, with the concentration of each AA in the protein drinks (R^2^ range 0.11–0.84) and also between each AA with gastric emptying (R^2^ range 0.16–0.71). Taken together, the current study extends these insights by demonstrating that factors other than the protein load and rate of gastric emptying of the protein test drinks influence the concentrations of specific AAs in the peripheral circulation. Such factors likely to account for the differences in concentrations of specific AAs reaching the peripheral circulation between studies may include the concentration of free AAs, as well as di- and tri-peptides within the source of protein (i.e., whether protein is intact, isolated, highly hydrolysed), differential digestion, metabolic transformation (which is particularly extensive for the dicarboxylic AAs, glutamic and aspartic acids) and absorption, of the exogenous and endogenous small intestinal proteins, and the rates of uptake and release of AAs from the liver and other tissues [31].

Evidence for differential roles of specific AAs in the regulation of glucose- and appetite-regulatory hormones, and the suppression of energy intake, is derived from studies in both animals [32,33] and humans [34,35,36,37,38]. In healthy men, we have reported previously that leucine [34], but not valine [35], each infused ID at a rate of 0.45 kcal/min for 90 min, modulated gut motor and hormone functions, blood glucose and/or energy intake. We also found that intragastric administration of lysine (at loads of 5 and 10 g) reduced the glycaemic response to a mixed-nutrient drink moderately [36]. Moreover, in lean and obese individuals, intragastric administration of 3 g of tryptophan slowed gastric emptying and increased glucagon, but did not suppress subsequent energy intake substantially [37], whereas a solution containing 3.3 g tryptophan infused ID at a rate of 0.15 kcal/min for 90 min, stimulated pyloric pressures, the release of CCK, and to a lesser extent, GLP-1 and PYY, and significantly reduced energy intake by ~200 kcal [38]. In contrast to the aforementioned studies, a strength of the current design was that we measured all 20 AAs and, thereby, could evaluate the interplay between these AAs and how they collectively influence glucose- and appetite-regulatory hormones, as well as energy intake. As such, the current study extends current knowledge [13,14,15,16,34,35,36,37,38] by demonstrating that leucine, valine, isoleucine, methionine and lysine (all essential AAs) and tyrosine (conditionally-essential AA) are each associated strongly with postprandial concentrations of CCK, GLP-1, insulin and glucagon, to a lesser extent with ghrelin, and weakly with protein-induced suppression of energy intake.

Notably, the blood glucose response was not related to the release of any AA. This was not surprising since the protein drinks contained no carbohydrate, and the almost immediate release of insulin (i.e., from ~15 min) was presumably counteracted by the release of glucagon from ~60 min following the drinks so that euglycaemia was maintained. These findings again highlight the strong association between AAs with the release of insulin, which is likely to reflect direct stimulation of pancreatic β-cells [39]. While plasma AA responses were also strongly, and positively, correlated with the glucagon response, whether its release is attributable to direct stimulation of the pancreatic α-cells by specific AAs remains uncertain. Interestingly, a study in rodents in which a high-protein, carbohydrate-free solution was perfused through pancreatic islet cells, found that arginine, glutamine and α-aminobutyrate stimulated glucagon secretion [40].

Several aspects of our study design should be considered when interpreting our results. Only healthy males were included, hence, our results may not reflect responses in women, overweight/obese or older individuals, although this is unlikely [41]. Although the 30 and 70 g loads of whey protein isolate used in this study contained different amounts of energy, our hypothesis was to investigate the effects of increasing protein loads that are typically consumed within drinks, snacks or main meals. As such, it cannot be assumed that our observations would extend to drinks that are isocaloric, or that contain fully hydrolysed form of proteins. Had we used a fully hydrolysed form of whey protein, the AAs may have been rapidly absorbed from the GI tract and may have differentially affected the responses we assessed [42]. Moreover, it cannot be assumed that our observations would extend to drinks containing other sources of protein, or a solid protein. For example, it has been reported that casein compared to whey, and solid compared to liquefied protein, are more slowly digested and absorbed [43,44]. However, evidence from a 2013 systemic review indicates that findings remain inconsistent regarding the protein kinetics from intact compared to hydrolysed forms of whey or casein, or of various protein sources [45]. Finally, we recognise that the relationships explored in this secondary analysis do not establish causality, and with no adjustment to *P*-values for multiple comparisons, our results are hypothesis-generating in nature. Hence, our findings dictate the need for further investigation to elucidate the role of these specific AAs in the regulation of glucose homeostasis and energy intake. A carefully designed, prospective study is clearly warranted to disentangle the effects of protein, different AAs and energy on correlations between postprandial responses of individual AAs and other study outcomes.

In conclusion, this study provided new insights into the concentrations of 20 AAs reaching the peripheral circulation following drinks containing loads of whey protein isolate that are representative of loads commonly consumed by humans. Our observations indicate that, in healthy, lean men, plasma concentrations of specific AAs (particularly the essential AAs) increase in a load-of-protein-dependent manner, and there are strong relationships between CCK, GLP-1, insulin and glucagon with leucine, isoleucine, valine, lysine, methionine, tryptophan, aspartic acid and tyrosine. In contrast, there was no relationship between blood glucose concentrations with plasma AAs, and relationships between ghrelin and energy intake with AAs were weak. Accordingly, our observations demonstrate that factors mediating the effects of dietary protein on blood glucose and energy intake are multifactorial and inter-related.

## Figures and Tables

**Table 1:** Baseline (fasting) plasma amino acid (AA) concentrations prior to consumption of the test drinks consisting of either 0 g (C), 30 g (L) or 70 g (H) of pure whey protein dissolved in varying amounts of distilled water, diet cordial, and sodium chloride (all 450 mL and 88 mOsm/L) ^a^.

Treatment					
AA	C	L	H	F_2,30_	*P Value* ^b^
mmol/L					
Gln (C)	0.80 ± 0.6	0.74 ± 0.07	0.81 ± 0.08	0.78	0.468
Ala (NE)	0.41 ± 0.03	0.38 ± 0.04	0.43 ± 0.04	1.41	0.261
Gly (C)	0.30 ± 0.02	0.28 ± 0.03	0.31 ± 0.03	1.15	0.329
Val (E)	0.28 ± 0.02	0.26 ± 0.02	0.29 ± 0.03	0.71	0.499
Pro (C)	0.26 ± 0.02	0.27 ± 0.04	0.27 ± 0.03	0.07	0.930
Lys (E)	0.20 ± 0.02	0.19 ± 0.02	0.21 ± 0.02	0.89	0.420
Leu (E)	0.14 ± 0.01	0.14 ± 0.01	0.15 ± 0.02	0.74	0.488
Thr (E)	0.14 ± 0.01	0.13 ± 0.01	0.14 ± 0.01	1.54	0.232
Ser (C)	0.12 ± 0.01	0.11 ± 0.01	0.12 ± 0.01	1.06	0.359
Arg (C)	0.10 ± 0.01	0.09 ± 0.01	0.10 ± 0.01	2.03	0.148
His (E)	0.10 ± 0.01	0.09 ± 0.01	0.10 ± 0.01	0.45	0.639
Ile (E)	0.08 ± 0.01	0.07 ± 0.01	0.08 ± 0.01	2.16	0.132
Tyr (C)	0.07 ± 0.01	0.07 ± 0.01	0.08 ± 0.01	1.53	0.234
Asn (NE)	0.07 ± 0.01	0.06 ± 0.01	0.07 ± 0.01	0.56	0.580
Phe (E)	0.06 ± 0.01	0.06 ± 0.01	0.06 ± 0.01	0.65	0.527
Glu (NE)	0.06 ± 0.01	0.05 ± 0.01	0.05 ± 0.01	1.12	0.341
Cys (C)	0.03 ± 0.01	0.03 ± 0.01	0.03 ± 0.01	0.16	0.850
Met (E)	0.03 ± 0.003	0.03 ± 0.003	0.03 ± 0.003	0.62	0.543
Trp (E)	0.005 ± 0.00	0.005 ± 0.00	0.006 ± 0.00	3.88	0.032
Asp (NE)	0.004 ± 0.00	0.004 ± 0.00	0.004 ± 0.00	1.94	0.162
Total	3.27 ± 0.21	3.06 ± 0.26	3.35 ± 0.28	0.94	0.404

^a^ Data are means ± SEMs, *n =* 16; amino acids (AAs) have been presented in order of highest to lowest concentration. ^b^ Main effect of protein load was determined by one-way repeated measures ANOVA; statistical significance was accepted at *p* < 0.05. Abbreviations for AAs: Alanine: Ala; Arginine: Arg; Asparagine: Asn; Aspartic Acid: Asp; Cysteine: Cys; Glutamine: Gln; Glutamic acid: Glu; Glycine: Gly; Histidine: His; Isoleucine: Ile; Leucine: Leu; Lysine: Lys; Methionine: Met; Phenylalanine: Phe; Serine: Ser; Threonine: Thr; Tryptophan: Trp; Tyrosine: Tyr Valine: Val. Essential AAs (E); Non-essential AAs (NE); Conditional AAs (C).

**Table 2:** Plasma amino acid (AA) responses (incremental areas under the curve (iAUC)_0–180 min_ displayed in order of abundance of the amino acids within the whey protein drinks) following consumption of the test drinks consisting of either 0 g (C), 30 g (L) or 70 g (H) of whey protein dissolved in varying amounts of distilled water, diet cordial, and sodium chloride (all 450 mL and 88 mOsm/L) ^a^.

Treatment					
AA	C	L	H	F_2,30_	*P Value* ^b^
mmol·180 min·L^−1^					
Glu (NE)	1.9 ± 0.4	8.1 ± 1.2 ^c^	11.2 ± 1.0 ^c,d^	36.8	<0.001
Leu (E)	0.6 ± 0.2	48.4 ± 3.5 ^c^	74.4 ± 5.2 ^c,d^	133	<0.001
Asp (NE)	0.1 ± 0.02	1.7 ± 0.2 ^c^	3.5 ± 0.3 ^c,d^	74.0	<0.001
Lys (E)	1.7 ± 0.4	33.7 ± 3.1 ^c^	49.6 ± 4.4 ^c,d^	67.6	<0.001
Ile (E)	0.3 ± 0.1	22.4 ± 1.7 ^c^	35.0 ± 2.5 ^c,d^	123	<0.001
Val (E)	1.5 ± 0.5	30.1 ± 3.3 ^c^	40.3 ± 4.1 ^c^	45.1	<0.001
Ala (NE)	2.3 ± 0.7	22.1 ± 3.0 ^c^	18.7 ± 3.0 ^c^	15.0	0.001
Thr (E)	1.1 ± 0.3	14.1 ± 1.5 ^c^	18.2 ± 2.0 ^c^	35.7	<0.001
Pro (C)	2.1 ± 0.6	15.3 ± 1.8 ^c^	14.1 ± 2.0 ^c^	16.1	<0.001
Tyr (C)	0.3 ± 0.1	8.4 ± 0.9 ^c^	12.7 ± 1.3 ^c,d^	51.6	<0.001
Ser (C)	0.9 ± 0.2	6.9 ± 1.0 ^c^	7.1 ± 1.0 ^c^	14.6	<0.001
Phe (E)	0.3 ± 0.1	3.9 ± 0.6 ^c^	5.3 ± 0.8 ^c^	22.6	<0.001
Cys (C)	0.3 ± 0.1	1.7 ± 0.4 ^c^	1.3 ± 0.3 ^c^	7.43	0.002
Arg (C)	0.5 ± 0.1	6.9 ± 0.9 ^c^	8.2 ± 1.0 ^c^	24.4	0.001
Met (E)	0.2 ± 0.05	4.2 ± 0.4 ^c^	6.6 ± 0.7 ^c,d^	52.7	<0.001
Trp (E)	0.04 ± 0.01	0.82 ± 0.07 ^c^	1.15 ± 0.1 ^c^	66.2	<0.001
His (E)	0.8 ± 0.2	3.9 ± 0.7 ^c^	2.8 ± 0.4 ^c^	8.13	0.006
Gly (C)	2.6 ± 0.6	6.3 ± 1.4	3.2 ± 0.8	3.50	0.066
Gln (C)	8.5 ± 1.9	25.4 ± 4.8 ^c^	14.9 ± 3.1	5.30	0.011
Asn (NE)	0.6 ± 0.1	5.9 ± 0.7 ^c^	6.9 ± 0.9 ^c^	23.9	<0.001
Total	23.2 ± 6.0	255.25 ± 29.0 ^c^	302.9 ± 34.0 ^c^	27.9	<0.001

^a^ Data are means ± SEMs, *n =* 16. ^b^ Main effect of treatment on the iAUC_0–180 min_ for individual AAs was determined by one-way repeated measures ANOVA and post-hoc comparisons between two loads were determined using Bonferroni’s correction; statistical significance was accepted at *p* < 0.05; ^c^ Significantly different from C (*p* < 0.05); ^d^ Significantly different from L (*p* < 0.05). Abbreviations for AAs: Alanine: Ala; Arginine: Arg; Asparagine: Asn; Aspartic Acid: Asp; Cysteine: Cys; Glutamine: Gln; Glutamic acid: Glu; Glycine: Gly; Histidine: His; Isoleucine: Ile; Leucine: Leu; Lysine: Lys; Methionine: Met; Phenylalanine: Phe; Serine: Ser; Threonine: Thr; Tryptophan: Trp; Tyrosine: Tyr; Valine: Val. Essential AAs (E); Non-essential AAs (NE); Conditional AAs (C).

## Appendix A

**Table A1:** Amino acid (AA) composition of test drinks containing 30 g (L) or 70 g (H) of pure whey protein isolate ^a,b^.

Treatment			
AA	L	H	Per 100 g
	g [mmol/L]	g [mmol/L]	g
Glu	5.7 [88]	13.2 [211]	18.9
Leu	4.3 [77]	10.1 [181]	14.4
Asp	4.0 [69]	9.2 [163]	13.2
Lys	3.8 [59]	8.8 [142]	12.5
Ile	2.0 [35]	4.6 [83]	6.6
Val	1.8 [35]	4.1 [82]	5.9
Ala	1.8 [46]	4.1 [108]	5.9
Thr	1.7 [33]	3.9 [77]	5.5
Pro	1.4 [28]	3.4 [70]	4.8
Tyr	1.3 [16]	3.0 [39]	4.3
Ser	1.2 [26]	2.9 [65]	4.1
Phe	1.2 [17]	2.8 [40]	4.0
Cys	1.2 [23]	2.8 [54]	4.0
Arg	0.9 [12]	2.2 [30]	3.1
Met	0.8 [12]	1.9 [30]	2.7
Trp	0.8 [9]	1.9 [22]	2.7
His	0.7 [10]	1.5 [23]	2.2
Gly	0.6 [18]	1.4 [44]	2.0
Gln		Not reported	
Asn		Not reported	
Total	35.04	81.76	116.8

^a^ The amino acid (AA) composition of ‘Whey Protein Isolate 8855’ (Fonterra Co-Operative Group Ltd., Auckland, New Zealand) was provided by Fonterra, and glutamine and asparagine were not reported in the product specifications because they contributed to concentrations of glutamic acid and aspartic acid, respectively. ^b^ AAs (g) are presented in order of most to least abundant within the test drink. Abbreviations for AAs: Alanine: Ala; Arginine: Arg; Asparagine: Asn; Aspartic Acid: Asp; Cysteine: Cys; Glutamine: Gln; Glutamic acid: Glu; Glycine: Gly; Histidine: His; Isoleucine: Ile; Leucine: Leu; Lysine: Lys; Methionine: Met; Phenylalanine: Phe; Serine: Ser; Threonine: Thr; Tryptophan: Trp; Tyrosine: Tyr; Valine: Val.

**Table A2:** Within-subject relationships between the iAUCs_0–180 min_ of each of the 20 amino acids (displayed in order of abundance of the amino acids within the whey protein drinks) with the load of protein, following consumption of the test the drinks containing either 0 g (C), 30 g (L) or 70 g (H) of pure whey protein dissolved in varying amounts of distilled water, diet cordial and sodium chloride (all 450 mL and 88 mOsm/L).

AA	R^2^ *Value*	*P Value*
mmol/L		
Glu (NE)	0.66	<0.001
Leu (E)	0.84	<0.001
Asp (NE)	0.83	<0.001
Lys (E)	0.76	<0.001
Ile (E)	0.84	<0.001
Val (E)	0.66	<0.001
Ala (NE)	0.26	0.003
Thr (E)	0.61	<0.001
Pro (C)	0.31	0.001
Tyr (C)	0.72	<0.001
Ser (C)	0.35	<0.001
Phe (E)	0.54	<0.001
Cys (C)	0.14	0.034
Arg (C)	0.51	<0.001
Met (E)	0.74	<0.001
Trp (E)	0.74	<0.001
His (E)	0.11	0.062
Gly (C)	0	0.874
Gln (C)	Not reported	
Asn (NE)	Not reported	

Data are within-subject R^2^ values for *n* = 16; Statistical significance was accepted at *p* < 0.05. Abbreviations for AAs: Alanine: Ala; Arginine: Arg; Asparagine: Asn; Aspartic Acid: Asp; Cysteine: Cys; Glutamine: Gln; Glutamic acid: Glu; Glycine: Gly; Histidine: His; Isoleucine: Ile; Leucine: Leu; Lysine: Lys; Methionine: Met; Phenylalanine: Phe; Serine: Ser; Threonine: Thr; Tryptophan: Trp; Tyrosine: Tyr; Valine: Val. Essential AAs (E); Non-essential AAs (NE); Conditional AAs (C).

**Table A3:** Plasma gut hormone responses (AUCs_0–180 min_), gastric emptying and the amount and total energy consumed at a buffet meal, in response to the test drinks containing either 0 g (C), 30 g (L) or 70 g (H) of pure whey protein dissolved in varying amounts of distilled water, diet cordial, and sodium chloride (all 450 mL and 88 mOsm/L) ^a^.

	C	L	H	*P Value* ^b^
Ghrelin (pmol/L 180 min·L^−1^)	80,372 ± 10,256	66,690 ± 8536	59,053 ± 7854	<0.01
CCK (pmol/L 180 min·L^−1^)	729 ± 102	1016 ± 131	1215 ± 119	<0.001
GLP-1 (pmol/L 180 min·L^−1^)	4564 ± 439	6018 ± 408	7247 ± 470	<0.001
Insulin (mU/L 180 min·L^−1^)	503 ± 63	1463 ± 175	2275 ± 300	<0.01
Glucagon (pmol/L 180 min·L^−1^)	3187 ± 158	5973 ± 308	8219 ± 376	<0.001
T_50_ (min)	12 ± 0.5	26 ± 3 ^c^	65 ± 9 ^c,d^	<0.001
Gastric emptying rate (kcal/min)	NA	2.6 ± 0.3	2.9 ± 0.3	>0.05
Blood glucose (mmol/L 180 min·L^−1^)	932 ± 16	921 ± 19	900 ± 13	>0.05
Energy intake (kcal)	1174 ± 91	1027 ± 81 ^c^	998 ± 71 ^c^	<0.01
Amount eaten (g)	1097 ± 78	1114 ± 112	1068 ± 69	>0.05

^a^ Data are means ± SEMs; *n* = 16 for gut hormones, blood glucose, energy intake, amount consumed, and gastric emptying. ^b^ Main effect of protein load determined by one-way repeated measures ANOVA and post-hoc comparisons between two loads were determined using Bonferroni’s correction; statistical significance was accepted at *p* < 0.05; ^c^ Significantly different from C (*p* < 0.05); ^d^ Significantly different from L (*p* < 0.05). Abbreviations: CCK, cholecystokinin; GLP-1, glucagon-like peptide 1; T_50,_ the time at which 50% of the drink had emptied from the stomach (T50) and which was used to calculate gastric emptying rate of each protein drink.
